# Supplementary material for: Understanding SNAP Recipient Characteristics to Guide Equitable Expansion of Nutrition Incentive Programs in Diverse Food Retail Settings
Source: Int J Environ Res Public Health. 2022 Apr 20;19(9):4977. doi: 10.3390/ijerph19094977 (PMC9101614; doi:10.3390/ijerph19094977)
Supplement: Supplementary file 1 [file ijerph-19-04977-s001.zip › ijerph-1596743-Supplementary S1.pdf]

Supplementary S1
Year 1 - Cross Sectional Survey

Participant ID

____

1. Please tell me all the places where you got food for your household over the past 12 months. I will list different options. Did you shop for or receive food from ...

- ☐ Supermarket (e.g. Dave's, Kroger, Giant Eagle)
- ☐ Supercenter (e.g. Wal-Mart, Target)
- ☐ Smaller grocery stores (Marc's, Aldi's, Save a Lot)
- ☐ Warehouse club (Sam's Club, BJs, Costco)
- ☐ Convenience store/Corner store
- ☐ Specialty store (e.g. ethnic stores, meat market, seafood market, green grocer, bakery)
- ☐ Drug/Pharmacy (e.g. CVS, Rite Aid, Walgreens)
- ☐ Dollar variety (Dollar General, Dollar Tree, Family Dollar)
- ☐ Farmers' market
- ☐ Food pantry/bank or shelter
- ☐ Mobile Pantry/Free Fruit and Vegetable Drop Off
- ☐ Church
- ☐ Friends or family
- ☐ Other, please specify
- ☐ Don't know (don't read)

1. a) Other, specify:

____

2. What is the name of the store where you got most of the food for your household over the past 12 months? Pick one.

____

3. Where is [store_name] located?

____

4. How often over the past 12 months did you go to [store_name] to get food for your household? You can answer in the amounts per day, week, month, or year.

- ◯ times per day
- ◯ times per week
- ◯ times per month
- ◯ times per year

4. a) Number of times.

____

5. How do you usually get to [store_name]?

☐ Drive own car
☐ Use someone else's car
☐ Someone else drives me
☐ Walk
☐ Bus
☐ Taxi or taxi service (i.e. Uber, Lyft)
☐ Ride bicycle
☐ Other (specify)
☐ Don't know/not sure (Do not read)
☐ Refused (Do not read)

5. a) Other, please specify

____

SNAP Experience

The next questions are about your experience with SNAP/EBT (sometimes called food stamps) and other food assistance benefits.

6. In the past 12 months, did you or anyone who lives in your household receive SNAP/EBT/Food Stamp benefits?

◯ Yes
◯ No

INTERVIEWER NOTE: If no to above, DISCONTINUE survey.

7. In the past 12 months, about how many months did you have SNAP/EBT/Food Stamp benefits?

◯ 1
◯ 2
◯ 3
◯ 4
◯ 5
◯ 6
◯ 7
◯ 8
◯ 9
◯ 10
◯ 11
◯ 12

8. On what date did you last receive your SNAP/EBT/Food Stamp benefits?

____

9. On what date do you expect to receive your next SNAP/EBT/Food Stamp benefits?

____

10. Last month, what was the value of all money received on your SNAP/EBT/Food Stamp card?

____

11. How many people does your SNAP/EBT/Food Stamp card benefit for?

____

11. a) Number of adults

____

11. b) Number of children

____

12. Over your lifetime, about how long have you been receiving SNAP/EBT/Food Stamp benefits? Please include all the times you were on SNAP/EBT/Food Stamps added together, but don't include any breaks/the times in between when you weren't on SNAP/EBT/Food Stamps.

◯ Less than a year
◯ 1-2 years
◯ 3- 4 years
◯ 5 or more years
◯ Don't know/Not sure (Do not read)
◯ Refused

13. In the past 12 months, did you or anyone who lives in your household receive WIC benefits?

◯ Yes
◯ No
◯ Don't know/Not sure (Do not read)
◯ Refused

Produce Perks Use

The next questions are about the Produce Perks Program. You received a flyer about this program from our team. This program provides extra money to buy fruits and vegetables for people shopping with SNAP/EBT/Food Stamps.

14. How did you first learn about the Produce Perks incentive program? [INTERVIEWER NOTE: Do not read list. Pick one option that is best]

◯ Learned from the research team
◯ Social service agency (e.g. SNAP, WIC, Medicaid)
◯ Community organization (e.g. recreation center, faith-based organization, school, health center)
◯ Print advertisement (e.g., poster, flyer, postcard, newspaper, billboard)
◯ Social media (e.g., Facebook, Twitter, Instagram)
◯ Word of mouth (e.g., friend, family, colleague)
◯ Driving by
◯ Other, specify

14. a) Other, specify:

____

15. Have you ever used the Produce Perks program before?

◯ Yes
◯ No
◯ Don't Know/Not sure (Do not read)
◯ Refused

16. Why haven't you tried to use the Produce Perks program? (Do not read list. Check all that apply.)

☐ Did not know about program
☐ Schedule did not permit use
☐ Store does not have desired produce
☐ Poor quality produce choices at store
☐ Food prices at the store
☐ Did not feel comfortable shopping at the store
☐ Too busy to cook food right now
☐ Don't like fruits and vegetables
☐ Foods preferred are not in season
☐ Store not convenient to access
☐ Forgot to try it out
☐ Other, specify

16. a) Other, specify:

____

17. How likely are you to use the Produce Perks program in the next 6 months?

◯ Not at all
◯ Very little chance
◯ Somewhat likely
◯ Very likely

18. On average, about how much money do you spend on fruits and vegetables during a typical month? This can be for all types of fruits and vegetables (fresh, frozen, canned).

____

19. What motivated you to use the Produce Perks program? (Do not read list. Check all that apply.)

☐ The program saves me money
☐ The program is easy to use
☐ The program is offered at a store close to home
☐ I was curious to see if the coupon actually worked
☐ The program allows me to buy more fruits and vegetables
☐ I have a medical issue that requires me to incorporate more fruits and vegetables into my diet; the program helps me do that
☐ Other, specify

19. a) Other, specify:

____

20. When was the first time you used the Produce Perks Program?

____

21. Where have you used the Produce Perks Program? [INTERVIEWER NOTE: If person says they have used it and cannot remember the name, begin listing out all locations within 10 miles. Include "don't know' option.]

____

22. Of these, which place did you use the Produce Perks for the first time? [INTERVIEWER NOTE: Is person says they have used it and cannot remember the name begin listing out all locations within 10 miles. Include "don't know" option.]

____

The next questions are about [location 1] where you received Produce Perks. [Repeat for all stores or farmers market]

23. How often over the past 6 months did you go to [location 1] to get food for your household? Record as one of the following.

◯ times per day
◯ times per week
◯ times per month
◯ times per 6-month period

23. a) Number of times.

____

24. How often do you purchase fruits and/or vegetables when you go to [name of store]?

◯ Never
◯ Rarely
◯ Sometimes
◯ Often
◯ All the time

25. During a typical month, about how much money do you spend on fruits and vegetables at [location 1] ? This can be for all types of fruits and vegetables (fresh, frozen, canned).

____

26. Over the past 6 months, how often did you receive Produce Perks at ____ [location 1]?

◯ times per day
◯ times per week
◯ times per month
◯ times per 6-month period

26. a) Number of times.

____

27. Over the past 6 months, about how much Produce Perks did you receive from [location 1]?

____

28. How many times over the past six months did you go to [location 1] just to redeem your Produce Perks coupon/tokens?

◯ times per day
◯ times per week
◯ times per month
◯ times per 6-month period

28. a) Number of times.

____

29. How much of the fruits and vegetables that you get at [location 1] is paid for using Produce Perks?

◯ None or almost none (0%)
◯ Less than half (25%)
◯ About half (50%
◯ More than half (75%)
◯ All or almost all (100%)
◯ Don't know/Not sure
◯ Refused

30. Did you redeem/use all of your Produce Perks coupons/tokens at ___ [location 1]?

◯ Yes
◯ No
◯ Don't know/Not sure
◯ Refused

31. Can you share more information about why you did not use all of your Produce Perks at __ [location 1]? [INTERVIEWER NOTE: Do not read list. Check all that apply.]

☐ Schedule did not permit use
☐ Store did not have desired produce
☐ Poor quality produce choices at store
☐ Food prices at the store
☐ Did not feel comfortable shopping at the store
☐ Too busy to cook food right now
☐ Don't like fruits and vegetables
☐ Foods preferred are not in season
☐ Store not convenient to access
☐ Forgot to use them
☐ Saving them for a holiday or event
☐ Other, specify

31. a) Other, specify:

____

32. Have you received Produce Perks at a second location?

◯ Yes
◯ No

33. How often over the past 6 months did you go to [location 2] to get food for your household? Record as one of the following.

◯ times per day
◯ times per week
◯ times per month
◯ times per 6-month period

33. a) Number of times.

____

34. How often do you purchase fruits and/or vegetables when you go to [name of store]?

◯ Never
◯ Rarely
◯ Sometimes
◯ Often
◯ All the time

35. During a typical month, about how much money do you spend on fruits and vegetables at [location 2] ? This can be for all types of fruits and vegetables (fresh, frozen, canned).

____

36. Over the past 6 months, how often did you receive Produce Perks at ____ [location 2]?

◯ times per day
◯ times per week
◯ times per month
◯ times per 6-month period

36. a) Number of times.

____

37. Over the past 6 months, about how much Produce Perks did you receive from [location 2]?

____

38. How many times over the past six months did you go to [location 2] just to redeem your Produce Perks coupon/tokens?

◯ times per day
◯ times per week
◯ times per month
◯ times per 6-month period

38. a) Number of times.

____

39. How much of the fruits and vegetables that you get at [location 2] is paid for using Produce Perks?

◯ None or almost none (0%)
◯ Less than half (25%)
◯ About half (50%
◯ More than half (75%)
◯ All or almost all (100%)
◯ Don't know/Not sure
◯ Refused

---

40. Did you redeem/use all of your Produce Perks coupons/tokens at ___ [location 2]?

◯ Yes
◯ No
◯ Don't know/Not sure
◯ Refused

---

41. Can you share more information about why you did not use all of your Produce Perks at __ [location 2]? [INTERVIEWER NOTE: Do not read list. Check all that apply.]

☐ Schedule did not permit use
☐ Store did not have desired produce
☐ Poor quality produce choices at store
☐ Food prices at the store
☐ Did not feel comfortable shopping at the store
☐ Too busy to cook food right now
☐ Don't like fruits and vegetables
☐ Foods preferred are not in season
☐ Store not convenient to access
☐ Forgot to use them
☐ Saving them for a holiday or event
☐ Other, specify

---

41. a) Other, specify:

____

---

42. Have you received Produce Perks at a third location?

◯ Yes
◯ No

---

43. How often over the past 6 months did you go to [location 3] to get food for your household? Record as one of the following.

◯ times per day
◯ times per week
◯ times per month
◯ times per 6-month period

---

43. a) Number of times.

____

44. How often do you purchase fruits and/or vegetables when you go to [name of store]?

◯ Never
◯ Rarely
◯ Sometimes
◯ Often
◯ All the time

45. During a typical month, about how much money do you spend on fruits and vegetables at [location 3] ? This can be for all types of fruits and vegetables (fresh, frozen, canned).

____

46. Over the past 6 months, how often did you receive Produce Perks at ____ [location 3]?

◯ times per day
◯ times per week
◯ times per month
◯ times per 6-month period

46. a) Number of times.

____

47. Over the past 6 months, about how much Produce Perks did you receive from [location 3]?

____

48. How many times over the past six months did you go to [location 3] just to redeem your Produce Perks coupon/tokens?

◯ times per day
◯ times per week
◯ times per month
◯ times per 6-month period

48. a) Number of times.

____

49. How much of the fruits and vegetables that you get at [location 3] is paid for using Produce Perks?

◯ None or almost none (0%)
◯ Less than half (25%)
◯ About half (50%
◯ More than half (75%)
◯ All or almost all (100%)
◯ Don't know/Not sure
◯ Refused

50. Did you redeem/use all of your Produce Perks coupons/tokens at ___ [location 3]?

◯ Yes
◯ No
◯ Don't know/Not sure
◯ Refused

51. Can you share more information about why you did not use all of your Produce Perks at __ [location 3]? [INTERVIEWER NOTE: Do not read list. Check all that apply.]

☐ Schedule did not permit use
☐ Store did not have desired produce
☐ Poor quality produce choices at store
☐ Food prices at the store
☐ Did not feel comfortable shopping at the store
☐ Too busy to cook food right now
☐ Don't like fruits and vegetables
☐ Foods preferred are not in season
☐ Store not convenient to access
☐ Forgot to use them
☐ Saving them for a holiday or event
☐ Other, specify

51. a) Other, specify:

____

At ___ [location 1], how often did you buy the following foods with Produce Perks?

	Never	Rarely	Sometimes	Always	Not Applicable
52. Fruits or vegetables that you had never tried or eaten before.	◯	◯	◯	◯	◯
53. Fruits or vegetables that your children had never tried or eaten before.	◯	◯	◯	◯	◯
54. Fruits or vegetables that are important to your culture.	◯	◯	◯	◯	◯
55. Fruits or vegetables that you typically buy.	◯	◯	◯	◯	◯
56. Fruits or vegetables recommended by your doctor or other health care provider.	◯	◯	◯	◯	◯
57. Fruits or vegetables that you normally skip because of costs.	◯	◯	◯	◯	◯

The next questions ask you to compare the farmers' market and grocery store in your area where Produce Perks is offered. You can answer these based on your best guess.

In your area, Produce Perks is offered at [name of store] and [name of farmers' market].

58. How do the prices of fresh fruits and vegetables at [name of store] compare to the prices of fresh fruits and vegetables at [name of farmers' market]? In general, would you say [name of store] has...

◯ A lot higher prices than [name of farmers' market]
◯ Somewhat higher prices than [name of farmers' market]
◯ About the same prices as [name of farmers' market]
◯ Somewhat lower prices than [name of farmers' market]
◯ A lot lower prices than [name of farmers' market]

59. How does the quality of fresh fruits and vegetables at [name of store] compare to the quality of fresh fruits and vegetables at [name of farmers' market]? In general, would you say [name of store] has...

◯ A lot worse quality than [name of farmers' market]
◯ Somewhat worse quality than [name of farmers' market]
◯ About the same quality as [name of farmers' market]
◯ Somewhat better quality than [name of farmers' market]
◯ A lot better quality than [name of farmers' market]

60. How does the variety of fresh fruits and vegetables at [name of store] compare to the variety of fresh fruits and vegetables at [name of farmers' market]? In general, would you say [name of store] has...

◯ A lot smaller variety than [name of farmers' market]
◯ Somewhat smaller variety than [name of farmers' market]
◯ About the same variety as [name of farmers' market]
◯ Somewhat larger variety than [name of farmers' market]
◯ A lot larger variety than [name of farmers' market]

61. How does the service at [name of store1] compare to the service of [name of farmers' market]? In general, would you say [name of store] has...

◯ A lot less welcoming and friendly service than [name of farmers' market]
◯ Somewhat less welcoming and friendly service than [name of farmers' market]
◯ About the same service as [name of farmers' market]
◯ Somewhat more welcoming and friendly service than [name of farmers' market]
◯ A lot more welcoming and friendly service than [name of farmers' market]

Social Connections and Food Shopping

For this question, think about the people who are close to you. This can be your friends, family, or co-workers.

	None	Less than half	About half	More than half	All	Don't know/Not sure	Refused
62. About how many shopped at ___ [name of store] over the past 6 months?	◯	◯	◯	◯	◯	◯	◯

63. About how many shopped at ___ [name of farmers' market] over the past 6 months?	◯	◯	◯	◯	◯	◯	◯
64. About how many have used Produce Perks over the past 6 months?	◯	◯	◯	◯	◯	◯	◯

Time Costs of Foods Purchased and Consumed

Please rate how much you agree or disagree with the following statements.

65. I never have enough time to shop for fruits and vegetables.

◯ Strongly Disagree
◯ Disagree
◯ Neutral
◯ Agree
◯ Strongly Agree

66. What is the main reason you felt that you did not have time to shop for fruits and vegetables?

◯ Transportation
◯ Lack of childcare
◯ Health status
◯ Other

66. a) Other, specify:

____

67. It is not convenient for me to buy fruits and vegetables.

◯ Strongly Disagree
◯ Disagree
◯ Neutral
◯ Agree
◯ Strongly Agree

68. What is the main reason you felt that it is not convenient for you to buy fruits and vegetables?

◯ Transportation
◯ Lack of childcare
◯ Health status
◯ Other

68. a) Other, specify:

____

69. I do not have time to cook.

◯ Strongly Disagree
◯ Disagree
◯ Neutral
◯ Agree
◯ Strongly Agree

70. What is the main reason you felt that you do not have time to cook?

◯ Transportation
◯ Lack of childcare
◯ Health status
◯ Other

70. a) Other, specify:

____

71. My food shopping is always rushed.

◯ Strongly Disagree
◯ Disagree
◯ Neutral
◯ Agree
◯ Strongly Agree

72. What is the main reason you felt that your food shopping is always rushed?

◯ Transportation
◯ Lack of childcare
◯ Health status
◯ Other

72. a) Other, specify:

____

Fruit and Vegetable Self Efficacy

Please rate the extent to which you feel confident about doing each of the following activities.

	Not at all confident	Not very confident	Neutral	Confident	Extremely confident
73. Preparing fresh green vegetables (e.g., broccoli, spinach).	◯	◯	◯	◯	◯
74. Preparing root vegetables (e.g., potatoes, beets, sweet potatoes).	◯	◯	◯	◯	◯

75. Preparing fruit (e.g., peaches, watermelon).	◯	◯	◯	◯	◯
76. Preparing fruits and vegetables with herbs and spices (e.g., basil, thyme, cayenne pepper).	◯	◯	◯	◯	◯

Dietary Outcomes

The next questions are about the fruits and vegetables you ate during the past 30 days (i.e., the past month). Please think about all forms of fruits and vegetables including cooked or raw, fresh, frozen or canned. Please think about all meals, snacks, and food consumed at home and away from home. I will be asking how often you ate or drank each one: for example, once a day, twice a week, three times a month, and so forth.

77. During the past month, how many times per day, week or month did you drink 100% PURE fruit juices? Do not include fruit-flavored drinks with added sugar or fruit juice you made at home and added sugar to. Only include 100% juice.

◯ Per day
◯ Per week
◯ Per month
◯ Never
◯ Don't know/Not sure
◯ Refused

77. a) Number of times.

____

78. During the past month, not counting juice, how many times per day, week, or month did you eat fruit? Count fresh, frozen, or canned fruit. Read only if necessary: "Your best guess is fine. Include apples, bananas, applesauce, oranges, grape fruit, fruit salad, watermelon, cantaloupe or musk melon, papaya, lychees, star fruit, pomegranates, mangos, grapes, and berries such as blueberries and strawberries."

INTERVIEWER NOTE: Do not count fruit jam, jelly, or fruit preserves. Do not include dried fruit in ready-to-eat cereals. Do include dried raisins, cran-raisins if respondent tells you - but due to their small serving size they are not included in the prompt. Do include cut up fresh, frozen, or canned fruit added to yogurt, cereal, jello, and other meal items.

◯ Per day
◯ Per week
◯ Per month
◯ Never
◯ Don't know/Not sure
◯ Refused

78. a) Number of times.

____

79. During the past month, how many times per day, week, or month did you eat cooked or canned beans, such as refried, baked, black, garbanzo beans, beans in soup, soybeans, edamame, tofu or lentils.

Do NOT include long green beans.

◯ Per day
◯ Per week
◯ Per month
◯ Never
◯ Don't know/Not sure
◯ Refused

79. a) Number of times.

____

80. During the past month, how many times per day, week, or month did you eat dark green vegetables for example broccoli or dark leafy greens including romaine, kale, collard greens or spinach?

Do not include iceberg lettuce if specifically stated.

◯ Per day
◯ Per week
◯ Per month
◯ Never
◯ Don't know/Not sure
◯ Refused

80. a) Number of times

____

81. During the past month, how many times per day, week, or month did you eat orange colored vegetables such as sweet potatoes, pumpkin,
winter squash, or carrots?

Do not include pumpkin bars, cake, bread or other grain-based dessert type food containing pumpkin (i.e. similar to banana bars, zucchini bars we do not include)

◯ Per day
◯ Per week
◯ Per month
◯ Never
◯ Don't know/Not sure
◯ Refused

81. a) Number of times.

____

82. Not counting what you just told me about, during the past month, about how many times per day, week, or month did you eat OTHER vegetables? Examples of other vegetables include tomatoes, tomato juice or V-8 juice, corn, eggplant, peas, lettuce, cabbage, and white potatoes that are NOT fried such as baked or mashed potatoes.

◯ Per day
◯ Per week
◯ Per month
◯ Never
◯ Don't know/Not sure
◯ Refused

82. a) Number of times.

____

Cooking/Eating Behaviors

83. How often do you cook meals made from scratch or using whole foods? (e.g., not frozen, boxed, or pre-prepared meals) [Interview note: Pick one time frame that best represents their pattern and record number.]

◯ Per day
◯ Per week
◯ Per month
◯ Per year
◯ Don't know/Not sure
◯ Refused

83. a) Number of times.

____

84. How often do you eat foods that were prepared away from the home in places such as restaurants, fast food places, food stands, or vending machines? (Include breakfast, lunch and dinner) [Interview note: Pick one time frame that best represents their pattern and record number.]

◯ Per day
◯ Per week
◯ Per month
◯ Per year
◯ Don't know/Not sure
◯ Refused

84. a) Number of times.

____

How much do you agree or disagree with the following statements:

	Strongly Disagree	Tend to Disagree	Tend to Agree	Strongly Agree	Don't Know
85. I am a healthy eater.	◯	◯	◯	◯	◯
86. I am someone who eats in a nutritious manner.	◯	◯	◯	◯	◯
87. I am someone who is careful about what I eat.	◯	◯	◯	◯	◯

Food Insecurity

Now I'm going to ask a few questions about your household.

88. How many people currently live in your household? Please include yourself. [Note: Include people who stay at the house most days of the week.]

____

88. a) How many of these people are adults 18 years or older?

____

88. b) How many of these people are children ages 0-17 years?

____

I'm going to read you several statements that people have made about their food situation. For these statements, please tell me whether the statement was often true, sometimes true, or never true for (you/your household) in the last 12 months. [INTERVIEWER NOTE: If one person in household, use "I' "my" and "you" in parentheticals, otherwise, use "we" "our" and "your household"]

89. The first statement is, "The food that (I/we) bought just didn't last, and (I/we) didn't have money to get more." Was that often, sometimes, or never true for (you/your household) over the last 12 months

◯ Often true
◯ Sometimes true
◯ Never true
◯ Don't Know
◯ Refused

90. "(I/we) couldn't afford to eat balanced meals." Was that often, sometimes, or never true for (you/your household) in the last 12 months?

◯ Often true
◯ Sometimes true
◯ Never true
◯ Don't Know
◯ Refused

91. In the last 12 months, did (you/you or other adults in your household) ever cut the size of your meals or skip meals because there wasn't enough money for food?

◯ Yes
◯ No
◯ Don't know/Not sure
◯ Refused

92. How often did this happen-almost every month, some months but not every month, or in only 1 or 2 months?

◯ Almost every month
◯ Some months but not every month
◯ Only 1 or 2 months
◯ Don't know/Not sure
◯ Refused

93. In the last 12 months, did you ever eat less than you felt you should because there wasn't enough money for food?

◯ Yes
◯ No
◯ Don't know/Not sure
◯ Refused

94. In the last 12 months, were you ever hungry but didn't eat because there wasn't enough money for food?

◯ Yes
◯ No
◯ Don't know/Not sure
◯ Refused

General Demographics

The next questions will help us better understand who took part in this survey.

95. What is your gender?

◯ Male
◯ Female
◯ Other
◯ Don't know/Not sure (do not read)
◯ Refused (do not read)

96. Are you Hispanic or Latino?

◯ Yes
◯ No
◯ Don't know/ Not sure (do not read)
◯ Refused (do not read)

97. Are you...

◯ Mexican, Mexican American, Chicano/a
◯ Puerto Rican
◯ Cuban
◯ Another Hispanic, Latino/a, or Spanish origin
◯ Don't know / Not sure (do not read)
◯ Refused (do not read)

98. What is your race? Note: Select all that apply

☐ White
☐ Black or African American
☐ American Indian or Alaska Native
☐ Asian
☐ Pacific Islander
☐ Other
☐ No additional choices (do not read)
☐ Don't know/not sure (do not read)
☐ Refused (do not read)

98. a) Other, specify:

____

99. Are you...

◯ Married
◯ Divorced
◯ Widowed
◯ Separated
◯ Never married
◯ A member of an unmarried or partnered couple (i.e., you live with someone but you are not married)
◯ Refused (Do not read)

100. What is your date of birth?

____

101. Do you own a car or have a vehicle you can freely use?

◯ Yes
◯ No
◯ Don't know
◯ Refused

102. What is the highest grade or year of school you completed? Read only if necessary:

◯ Never attended school or only attended kindergarten
◯ Grades 1 through 8 (Elementary)
◯ Grades 9 through 11 (Some high school)
◯ Grade 12 or GED (High school graduate)
◯ College 1 year to 3 years (Some college or technical school)
◯ College 4 years or more (College graduate)
◯ Post-graduate training but no degree
◯ Post-graduate degree (MS/MA, PhD, JD, MD)
◯ Refused (don't read)

103. In the last 12 months, what was your annual household income from all sources? (INTERVIEWER: If participant gives a range, add the halfway point of the range. i.e.: participant states $10,000-15,000, enter $12,500)

____

104. How often did you move or change addresses over the past 12 months? Record number:

____

105. How long have you stayed or lived at your current address? You can answer in amount of days, month, or years...

◯ Months
◯ Years
◯ Days

105. a) Number

____

106. Do you currently own or rent your home?

◯ Own
◯ Rent
◯ Other arrangement
◯ Don't know/Not sure (don't read)
◯ Refused (don't read)

Health Status

107. Would you say that in general your health is...

◯ Excellent
◯ Very Good
◯ Good
◯ Fair
◯ Poor
◯ Don't know/Not sure
◯ Refused

Employment

At the time of your original survey completion date (provide survey completion date) were you OR are you currently....?

◯ Employed for wages
◯ Self-employed
◯ Out of work for 1 year or more
◯ Out of work for less than 1 year
◯ A homemaker
◯ A Student
◯ Retired
◯ Unable to work
◯ Refused (don't read)

That was our last question. Do you have any questions before we wrap up today?

We will mail your gift card within the next 7 business days. We will mail the gift card to the following address [street], [city], [state], [zipcode]. Is this okay? Please make sure this is a reliable address, we are not responsible for any lost or stolen gift cards.

If this is not the correct address, please tell us where you would like your gift card mailed to.

Date survey was completed

____

FOR RESEARCH STAFF TO COMPLETE AFTER SURVEY
refer to response of question 21 to answer this question

Where has this participant used the Produce Perks program?

◯ ONLY at a farmer's market
◯ ONLY at a retail site
◯ BOTH farmer's market and retail site
◯ None of the above
