# Supplementary material for: Understanding SNAP Recipient Characteristics to Guide Equitable Expansion of Nutrition Incentive Programs in Diverse Food Retail Settings
Source: Int J Environ Res Public Health. 2022 Apr 20;19(9):4977. doi: 10.3390/ijerph19094977 (PMC9101614; doi:10.3390/ijerph19094977)
Supplement: Supplementary file 1 [file ijerph-19-04977-s001.zip › ijerph-1596743-Table S1.pdf]

Supplementary Materials

Table S1. Participant Characteristics Tested for Differences by Produce Perks Use Status

	P-value for difference	
	**Nutrition Incentive Program Users Vs. Non-Users**	**Nutrition Incentive Program by Location of Use<br><br>Retail vs. Farmer's Market vs. Both**
**Economic Domain**		
Annual Income in dollars	<0.001 [a]	0.685 [b]
Number of people supported by your SNAP	0.005 [a]	0.332 [b]
Amount of SNAP money received last month	0.039 [a]	0.152 [b]
Total years on SNAP (lifetime)	0.848 [a]	0.477 [b]
Someone in household received WIC in the last year	0.896 [c]	0.589 [d]
Amount spent on fruits and vegetables during a typical month	0.593 [a]	0.132 [b]
Over the past 6 months, about how much Produce Perks did you receive from the location of first use?	NA	0.030 [b]
You had at least one month in the past year where you did not receive SNAP	0.109 [c]	0.508 [d]
**Spatial Temporal Domain**		
It is not convenient to buy fruits and vegetables (1 = strongly disagree, 5 = strongly agree)	0.005 [a]	0.339 [b]
Distance to preferred store	0.020 [a]	0.469 [b]
How do you usually get to your food store/market/pantry		
Have a car	0.051 [c]	0.799 [d]
Use someone else's car	0.048 [d]	0.468 [d]
Someone drives me	0.459 [c]	0.605 [d]
Walk	0.716 [c]	0.323 [d]
Take a bus	0.409 [c]	0.280 [d]
Taxi or taxi service (i.e. Uber, Lyft)	0.588 [d]	1.000 [d]

Ride a bicycle	0.131 [d]	0.297 [d]
Other	0.756 [d]	0.218 [d]
I never have enough time to shop for fruits and vegetables (1 = strongly disagree, 5 = strongly agree)	0.159 [a]	0.548 [b]
In the last year I obtained food from a		
Supermarket (e.g. Dave's, Kroger, Giant Eagle)	0.229 [d]	NA
Supercenter (e.g. Wal-Mart, Target))	0.501 [c]	NA
Smaller grocery store (e.g. Marcs, Aldi, Save a Lot)	0.796 [d]	NA
Warehouse club (e.g. Sam's Club, BJs, Costco)	0.598 [c]	NA
Convenience store/Corner store	0.567 [c]	NA
Specialty store (e.g. ethnic stores, bakery, meat market, seafood market, green grocer)	0.038 [c]	NA
Drug Store/Pharmacy (e.g. CVS, Rite Aid, Walgreens)	0.207 [c]	NA
Dollar variety (e.g. Dollar General, Dollar Tree, Family Dollar)	0.540 [c]	NA
Farmer's market	0.034 [c]	NA
Food pantry/bank or shelter	0.731 [c]	NA
Mobile Pantry/Free Fruit and Vegetable Drop Off	0.044 [c]	NA
Church	0.543 [c]	NA
Friends or Family	0.134 [c]	NA
Another option not listed here	0.519 [d]	NA
Number of visits per month to your primary store	0.597 [a]	0.009 [b]
**Service Domain**		
How do the prices of fresh fruits and vegetables at your primary store compare to the prices of fresh fruits and vegetables at the farmers' market? (1 = a lot higher, 5 = a lot lower)	0.477 [a]	0.609 [b]
How does the quality of fresh fruits and vegetables at your primary store compare to the quality of fresh fruits and vegetables at the farmers' market? (1 = a lot worse, 5 = a lot better)	0.168 [a]	0.201 [b]

How does the variety of fresh fruits and vegetables at your primary store compare to the variety of fresh fruits and vegetables at the farmer's market? (1 = a lot larger variety, 5 = a lot smaller variety)	0.184 [a]	< 0.001 [b]
How does the service at your primary store compare to the service at the farmer's market? (1 = a lot less welcoming and friendly service, 5 = a lot more welcoming and friendly service)	0.694 [a]	0.411 [b]
**Social Domain**		
Think about friends, family, or people you know...		
About how many shopped at the promoted store offering Produce Perks over the past 6 months? (1=none, 5=all)	0.682 [a]	0.054 [b]
About how many have used Produce Perks over the past 6 months? (1=none, 5=all)	<0.001 [a]	0.225 [b]
About how many have shopped at your farmers' market over the past 6 months? (1=none, 5=all)	0.054 [a]	0.091 [b]
**Personal Domain**		
Education (Some high school or less, High school graduate, or Some college or more)	0.061 [a]	0.025 [b]
Moved in the last 12 months	0.050 [c]	1.000 [d]
Number of years you have been at your current address	0.213 [a]	0.958 [b]
Food Insecurity (USDA 6 item food security score > 1, indicating low or very low food security)	0.044 [c]	0.703 [d]
Food Insecurity (Alternative Ordinal specification: 0 = high food security, 6 = very low food security)	0.100 [a]	0.577 [b]
How often do you purchase fruits and vegetables when you go to your primary store? (1=never 5=always)	NA	<0.001 [b]
Confidence in preparing fresh green vegetables (1 = not at all confident, 5 = extremely confident)	0.273 [a]	0.008 [b]
Confidence in preparing root vegetables (1 = not at all confident, 5 = extremely confident)	0.435 [a]	0.018 [b]
Confidence in preparing fruit (1 = not at all confident, 5 = extremely confident)	0.426 [a]	0.185 [b]

Confidence in preparing fruits and vegetables with herbs and spices (1 = not at all confident, 5 = extremely confident)	0.430 [a]	0.153 [b]
I am a healthy eater (1 = Strongly Disagree, 4 = Strongly Agree)	0.549 [a]	0.454 [b]
I am someone who eats in a nutritious manner (1 = Strongly Disagree, 4 = Strongly Agree)	0.161 [a]	0.600 [b]
I am someone who is careful about what I eat (1 = Strongly Disagree, 4 = Strongly Agree)	0.148 [a]	0.522 [b]
Total number of people in your household	0.086 [a]	0.196 [b]
Number of adults in your household	0.870 [a]	0.067 [b]
Number of children in your household	0.034 [a]	0.107 [b]
Married or have a living partner	0.623 [c]	0.352 [d]
Age in Years	0.371 [a]	0.038 [b]
Home (Rent vs. Own vs. Other arrangement)	0.873 [c]	0.125 [d]
Race		
White	0.552 [c]	0.503 [d]
Black	0.942 [c]	0.056 [d]
Other	0.395 [d]	0.520 [d]
Gender	0.194 [c]	0.530 [d]
Self-reported health status (1= excellent, 5 = poor)	0.516 [a]	0.120 [b]
Number of months since first Produce Perks use	NA	<0.001 [a]
What motivated you to use the Produce Perks program?		
The program saves me money	NA	0.855 [d]
The program is easy to use	NA	1.000 [d]
The program is offered at a store close to home	NA	0.810 [d]
I was curious to see if the coupon actually worked	NA	0.509 [d]
The program allows me to buy more fruits and vegetables)	NA	0.164 [d]

I have a medical issue that requires me to incorporate more fruits and vegetables into my diet; the program helps me do that	NA	1.000 [d]
Other	NA	0.532 [d]
Number of times per month had fresh juice	0.893 [a]	0.170 [b]
Number of times per month ate fruit	0.613 [a]	0.631 [b]
Number of times per month ate beans	0.148 [a]	0.062 [b]
Number of times per month ate dark green vegetables	0.461 [a]	0.448 [b]
Number of times per month ate orange vegetables	0.669 [a]	0.287 [b]
Number of times per month ate "other" vegetables (beyond beans, dark green, or orange vegetables)	0.168 [a]	0.007 [b]
Number of times per month cooked meals made from scratch or using whole foods	0.259 [a]	0.076 [b]
Number of times per month ate out	0.359 [a]	0.437 [b]

[a] Wilcoxon-Mann-Whitney test (non-parametric analog of the t-test, assumes ordinal rather than normally distributed interval) [b] Kruskal Wallace (non-parametric analog of ANOVA, assumes ordinal rather than normally distributed interval) [c] Chi-Square test [d] Fischer's exact test (non-parametric analog of chi-squared test)
